# Poling of Glasses Using Resistive Barrier Discharge Plasma

**DOI:** 10.3390/ma15238620

**Published:** 2022-12-02

**Authors:** Sergey A. Scherbak, Vladimir P. Kaasik, Valentina V. Zhurikhina, Andrey A. Lipovskii

**Affiliations:** Higher School of Fundamental Physical Research, Peter the Great St. Petersburg Polytechnic University, 195251 St. Petersburg, Russia

**Keywords:** glass poling, open-anode, resistive barrier discharge, second harmonic generation, polarization current, ion mobilities

## Abstract

A technique for poling of glasses using a resistive barrier discharge plasma in the atmosphere in a gap of hundreds of microns is presented. Measurements of the polarization current, second harmonic generation, and IR spectra of poled soda-lime glass slides show that voltage sufficient to ignite plasma discharge provides efficient poling, whereas for lower voltages the poling effect is close to zero. We attributed this to the large number of hydrogen/hydronium ions generated from atmospheric water vapor by the plasma discharge in the gap, which penetrate into the glass. We also developed a simple model of poling according to Ohm’s law, analyzed the temporal dependencies of the polarization current and, basing on the model, estimated mobilities of hydrogen/hydronium and sodium ions in the glass: *μ*_H_ = (2.4 ± 0.8) × 10^−18^ m^2^V^−1^s^−1^ and *μ*_Na_ = (4.8 ± 1.8) × 10^−15^ m^2^V^−1^s^−1^. The values obtained are very close to the known literature data.

## 1. Introduction

The process of thermo-electric modification of glasses, i.e., glass poling, has been known for decades. In thermo-electric poling, mm-scale thick glass plates are heated and subjected to a DC potential. This procedure is very similar to the well-known charging of glass electrets, which, however, does not require heating [1,2], and, similarly to the charging of electrets, results in the accumulation of electric charge [3,4]. The heating allows for ionic transport in glasses in accordance with the Arrhenius law, whereas DC potential causes the drift of ions contained in glass. In this process, the subanodic region of the glass becomes depleted of mobile positive ions (or some kinds of them depending on the poling regime and glass composition) [5,6] which is called a depleted or a poled layer. Further cooling of the specimen, still under the voltage applied, “freezes” the formed distribution of ions, providing a highly stable structure capable of numerous applications. Particularly, poling with profiled electrodes can be used to fabricate structures for diffraction optics [7], since poled and initial glasses have different indices. Similarly, specific profiles and patterns can be made on glass surface via etching [8,9] or ion exchange [10] because poled and unpoled regions of glass are affected differently by etchants and differ in their diffusion properties [11]. Most importantly, poling breaks isotropy of glasses, adding a dedicated direction (direction of the applied electric field); therefore, poled glasses provide significant second order optical nonlinearity (SON), allowing for optical second harmonic generation (SHG) and sum frequency generation. Most often, SHG is associated with the third-order optical nonlinearity effect in the presence of a “frozen” DC electric field generated by the distribution of ions formed in thermal poling [12]. However, one cannot rule out such a reason for the appearance of SON as the orientation of dipolar entities in poled glass [13].

Glass poling can be carried out either in closed anode (without access of any species capable of penetrating the glass from atmosphere or other environment to the anodic surface of the glass, e.g., in vacuum, argon or using deposited film electrodes) [14] or in open-anode configuration which allows for access to the environment (mainly, atmosphere) to the anodic surface of the glass [15]. Moreover, there is an intermediate regime, when only a limited access of atmosphere is provided, e.g., pressed anode configuration [5]. Essentially, results of open-anode poling strongly depend on atmospheric species penetrating into the subanodic region of the glass [15,16]. One of the standard open-anode poling configurations is the so-called corona-poling with a needle-shaped anode [17]. Under a high voltage applied to the needle, plasma discharge between the anode and the specimen surface provides a large number of hydrogen/hydronium ions generated from atmospheric water vapor [18]. These ions penetrate into the glass, drift, and mostly compensate negative spatial charge left in the cation-depleted layer.

In this work, we demonstrate thermal poling configuration, which uses a small (0.2-mm-thick) gap between the anode and the specimen surface. Applying sufficiently high voltage results in a resistive (because of thermal activation of the glass conductivity) barrier DC plasma discharge in the gap [19], which generates hydrogen/hydronium ions penetrating into the glass, the number of which is less for lower voltages. Moreover, we show the effect of such poling on glasses via measurements of the passed charge and optical SHG.

## 2. Materials and Methods

In this study, we used microscope glass slides Menzel with high sodium content. The composition is (in wt.%) [20]: 72.20% SiO_2_, 14.30% Na_2_O, 6.40% CaO, 4.30% MgO, 1.20% K_2_O, 1.20% Al_2_O_3_, 0.30% SO_3_, and 0.03% Fe_2_O_3_. Particularly, atomic concentration of Na^+^ ions capable of drifting in the glass towards cathode is ~6.9∙10^27^ m^−3^. In our case, this is the only concentration of interest, since multivalent ions (Ca^2+^, Mg^2+^, Al^3+^, Fe^3+^) are less mobile than hydrogen/hydronium coming from atmosphere and, therefore, do not participate in the poling process. Potassium ions, K^+^, though, are involved in the drift [21], however, their concentration is almost 20 times lower than the one of Na^+^ and, therefore, they can be neglected.

**Figure 1:**
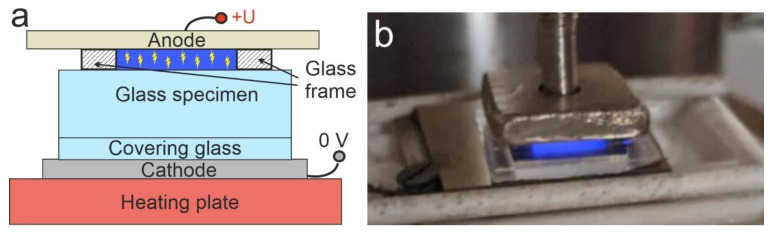
Scheme (**a**) and an actual photo (**b**) of the poling setup with glowing plasma under 1300 V applied.

In Figure 1a, we present the scheme of the plasma poling process described above. There, we designated a heating plate, a pressed cathode, a specimen, an anode, and a dielectric (glass) frame that provides 200 µm gap between the anode and the specimen. The poled region is ~8 × 8 mm^2^. Moreover, we used a thin (200 µm) plate of covering glass at the cathodic side of the specimen to prevent the formation of dendritic structures [22], which disturb SHG measurements. With a covering glass, dendrites form there and the cathodic side of the specimen remains clean after the covering glass is removed. In Figure 1b, we demonstrate a photo of the setup with light emission of plasma [23,24] in the gap under 1300 V applied. This blue glowing is associated with emission of N_2_ molecules [25]. We established that a voltage of about 1100 V is a characteristic voltage of plasma discharge formation in this 200 µm gap configuration (“plasma voltage”). However, we should note that it may depend on atmospheric conditions and the quality of the electrodes. Our experiments were carried out at room temperature 22 °C and relative humidity of the air 35%. This corresponds to ~0.6 wt.% water vapors in the air, which also contains ~75 wt.% N_2_, ~23 wt.%. O_2_, ~1 wt.% Ar and minor impurities. Moreover, for other kinds of glasses, the setup might need to be modified. Particularly, higher resistive glasses (e.g., glasses with lower sodium content or even alkali free, such as BF16 glass [26]) require higher voltages, so a modification of the glass frame may be necessary to avoid breakdown.

During poling, we measured the polarization current using Multimeter APPA109N (APPA Technology Corp., Taipei, Taiwan). We used the Maker fringes (MF) technique [27] for measurements of the second harmonic (SH) signal generated by poled glasses. This was first reported by Okada [17] in 1993. Here, we applied the same methodology to glasses poled using resistive barrier plasma discharge. The optical setup used was described in detail elsewhere [13]. A Nd:YAG laser (Litron, Rugby, UK) with a pulse duration of 6 ns and a wavelength of 1064 nm was used in the experiments. The essence of the method is to measure the dependence of the transmitted SH signal on the angle of the fundamental beam incidence. The fringes demonstrate the interference between SH signals generated by the poled (subanodic) region of the glass (poling-induced SON) and the cathodic surface of the glass, which possess SON conventional for any surface [28]. Thus, the resulting curves make it possible to compare these two SONs and to estimate the optical nonlinearity in the poled region. We used FTIR spectrometer FSM 2201 (Infraspek Ltd., Saint-Petersburg, Russia) to measure IR absorption around 3000–3500 cm^−1^, related to hydrogenated species penetrating into the glass during the poling.

## 3. Results and Discussion

### 3.1. Experimental

**Figure 2:**
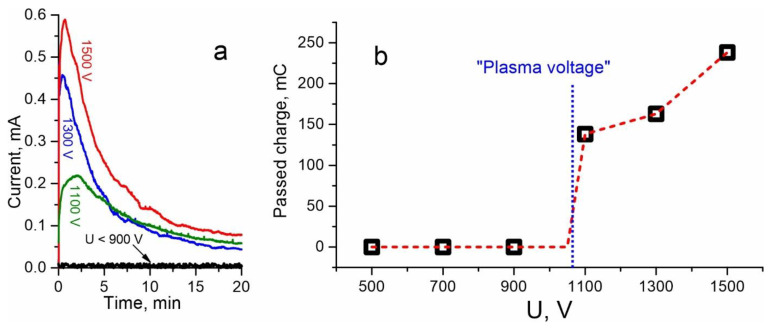
(**a**) Time dependencies of the current during the poling process of Menzel glass specimens subjected to different voltages. (**b**) Dependence of charge passed during 20-min-poling on the voltage applied (dashed curve is a guide for an eye); the characteristic ignition voltage of plasma (“plasma voltage”) is indicated.

We carried out a series of poling experiments with Menzel slides, varying the applied voltage and keeping the poling time constant (20 min plus 10 min for cooling of a specimen). Specimens were heated up to 250 °C. Time dependences of the current passing through the samples are presented in Figure 2a, and the overall charges passed through the samples during the process vs. applied voltage are shown in Figure 2b. The latter was obtained via integrating the curves presented in Figure 2a. For voltages 500–900 V (and, evidently, lower), which are below the “plasma voltage”, the current was negligible—see the lowest curve in Figure 2a which is indistinguishable from noise. For voltage 1100 V, which is sufficient to ignite plasma that is visible to the eye (see Figure 1b), the current and, respectively, the overall passed charge abruptly increase. This indicates an intense poling process. For higher voltages (1300 and 1500 V) passed charge monotonously increases, as expected. Note that after several minutes of poling, the light emission disappears, i.e., the discharge changes its type to the so-called “dark discharge” [29], though the current remains gradually decreasing. The fact that the poling carries on means that there is still plenty of hydrogen/hydronium ions in the environment of the specimen at this stage.

Note that relatively high passed charge (hundreds of mC) essentially exceeds the charge “frozen” in the samples. The latter is the difference of total positive charge of hydronium/hydrogen ions injected into the anode side of the glass and sodium ions ejected from the cathode side of the glass. This charge remains uncompensated after the poling. In our recent work, we showed that the “frozen” charge occupies only a thin area at the front of the depleted layer [30].

**Figure 3:**
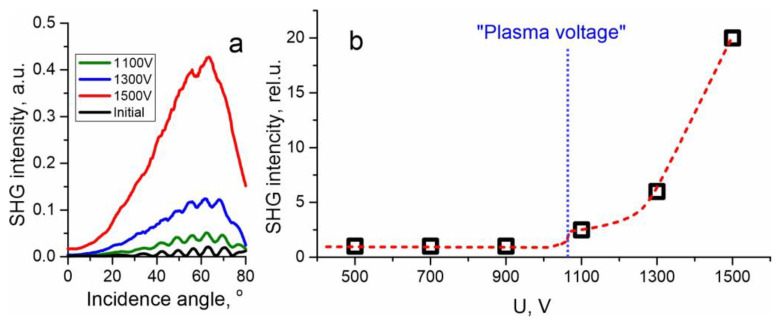
(**a**) Maker fringes for an initial glass (black) and for specimens subjected to 1100 V (olive), 1300 V (blue) and 1500 V (red). (**b**) Dependence of the maximal SH signal (at 63° incidence angle) on the voltage applied (dotted line is no more than guide for eyes); the dependence normalized by SH signal from the initial glass; ignition voltage of plasma is indicated.

Moreover, we measured SHG signal provided by the poled samples. In Figure 3a we show MF for a virgin Menzel glass and for specimens of this glass subjected to 1100 V, 1300 V and 1500 V. MF for voltages below the “plasma voltage” (~1070 V in our configuration) are similar to the MF for the virgin glass. For the virgin glass or glasses poled with insufficient voltage, the interference pattern in minima drops almost to zero (see black curve in Figure 3a) that is complete destructive interference—SONs on both sides of the sample, anodic and cathodic, coincide. In the case of a poled glass, subanodic layer of the specimen possesses a strong SON, whereas the SON of the cathodic surface stays the same. Therefore, the fringes minima are noticeably above zero for 1100 V (see olive curve in Figure 3a). For higher voltages of poling, induced SON is even higher and interference oscillations are barely seen (see blue and red curves in Figure 3a. In Figure 3b we plot maximal SH signal which corresponds approximately to 63° incidence angle of the excitation [31] vs. poling voltage.

The dependence in Figure 3b is qualitatively similar to the charge dependence in Figure 2b. We can make out two regions in these dependencies. The first is below the “plasma voltage”, where almost nothing happens to the specimens. We attribute this to the fact that without access of positive ions from atmosphere or with a limited access, there are no or insufficient charge compensation mechanisms. Therefore, electric field of uncompensated volume charge, which is directed opposite to the field created by the applied potential, rapidly stops the poling. In addition, before the ignition of the plasma, the resistance of the air gap between the glass and the anode electrode is high, and the voltage applied to the glass slide is less than after the ignition, when almost all of the voltage is applied to the slide due to the low resistance of the plasma. The latter situation seems to be similar to poling in vacuum under a moderate voltage, which also results in no SHG enhancement in the Menzel glass that we recently demonstrated [13]. Note that even without access of positive ions from atmosphere, other charge compensation mechanisms may occur, e.g., restructuring of glass matrix with the release of molecular oxygen and electrons [32], although this is possible under voltages higher than discussed. The second region, above the “plasma voltage”—where intensive poling occurs in the open-anode configuration—corresponds to a progressive increase in the current passing through the sample and the second harmonic signal with increasing voltage. It is worth noting that known models describing poling with an open-anode, e.g., the latest one by Oven [33], tacitly imply that there is a plenty of ions in atmosphere, and therefore provide monotonous dependencies of charge passed on the voltage applied. However, in experiments, the concentration of ions capable of entering the glass in the atmosphere should be taken into account. 

Thus, we experimentally showed that the simple access of the atmosphere to the anode does not necessarily provide open-anode configuration of poling, since the concentration of positive ions in the atmosphere may be insufficient for the effective charge compensation. “Physical” openness of the anode requires more conditions, e.g., voltage high enough to ignite plasma discharge that provides a large number of positive ions capable of penetrating into the glass.

**Figure 4:**
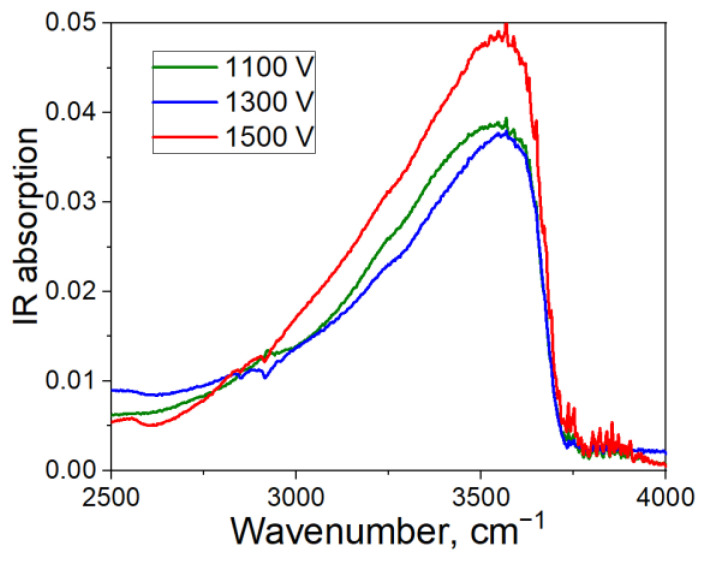
Differential (minus the spectrum of the initial glass) IR spectra of the specimens poled under 1100, 1300 and 1500 V.

We also measured the infra-red (IR) spectra of the poled specimens. The range of wavenumbers that is of particular interest is about 3000–3500 cm^−1^, where absorption peaks of water and differently bonded hydroxyl groups (“water” peaks) lye [34]. In Figure 4, we present the IR spectra of the specimens poled under 1100, 1300 and 1500 V (the IR spectrum of the virgin glass was subtracted). We observe a strong peak of “water” absorption around ~3500 cm^−1^ which supports the wide-spread statement that hydronium-like ions penetrate the glass during poling. Moreover, the specimen poled under 1500 V has the highest peak, whereas peaks for specimens poled under 1100 V and 1300 V are weaker. This correlates with the results of charge and SHG measurements which are the highest for the 1500 V-sample, and moderate for the others.

### 3.2. Modeling

**Figure 5:**
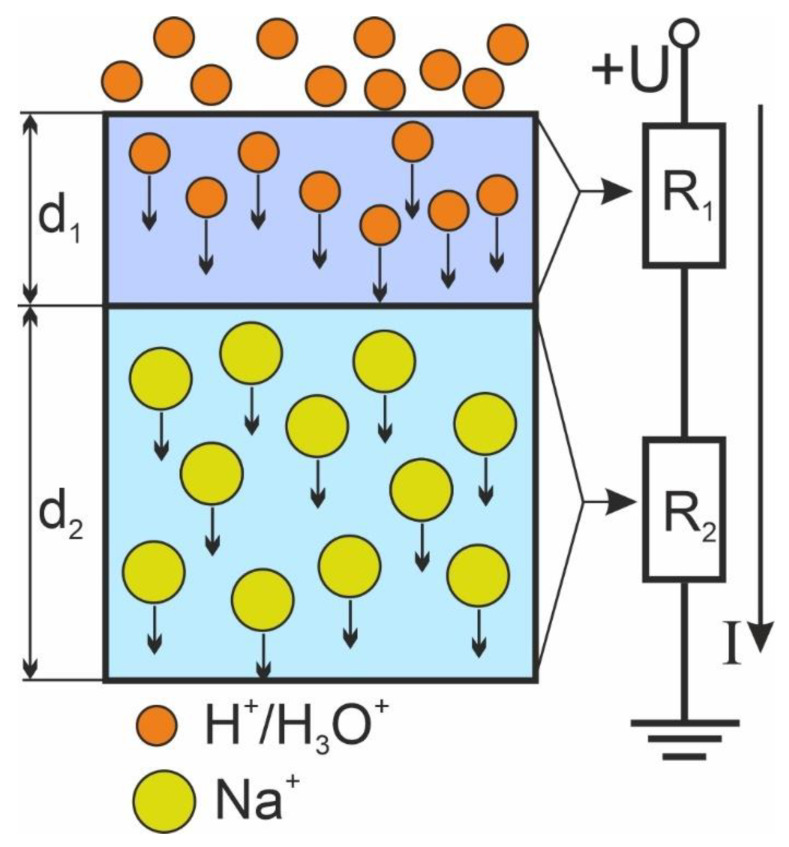
Schematic illustration of the poling process (**left**) and equivalent circuit (**right**). The poling illustration is not to actual scale.

In the model developed, we consider the poling process as a current flow through an equivalent circuit of two series resistances: *R*_1_ and *R*_2_, which correspond, respectively, to poled and unpoled regions of the glass. A schematic illustration of poling process and the equivalent circuit are shown in Figure 5. The poling illustration is not to actual scale for the sake of visual clarity. We supposed that the charge compensation is complete that is so-called local charge neutrality assumption [33]. This assumption is legit, since the “frozen” charge occupies only a very thin layer relatively to all volume of poled region and can be neglected.

The current *I* in the equivalent circuit when a constant DC voltage *U* is applied (we neglect voltage drop at the atmospheric plasma after the ignition) is:

(1) $$I(R_{1}+R_{2})=U$$

where *R*_1,2_ are:

(2) $$R_{1,2}=\frac{\rho_{1,2}d_{1,2}}{S}.$$

Here, *ρ*_1,2_ are resistivities, which depend on mobilities of ions in the corresponding regions, *S* is the samples lateral area, *d*_1,2_ are thicknesses of the regions. Due to micron-scale depth of poling *d*_1_, one can consider the thickness of the unpoled region *d*_2_ constant and equal to the thickness of the sample, whereas thickness of the poled region *d*_1_ depends on the total charge passed as follows:

(3) $$d_{1}(t)=\frac{1}{eC_{0}S}Q(t)=\frac{1}{eC_{0}S}\int_{0}^{t}I(t)dt,$$

where *e* is the fundamental charge and *C*_0_ is the initial concentration of mobile positive ions in the glass. Equation (3) directly follows from an expression for the total charge that occupies volume *d*_1_*S*. Equation (3) is valid when the front of the poling is plane and when only two kinds of ions are considered: one initially presents in the glass, the other penetrates into the glass from the environment.

From Equation (1) using above equations and introducing current via the charge passed it follows that:

(4) $$\frac{dQ(t)}{dt}\left(\frac{\rho_{1}}{S^{2}eC_{0}}Q(t)+R_{2}\right)=U.$$

Separating variables in Equation (4) we obtain the following solution:

(5) $$Q(t)=-\frac{R_{2}S^{2}eC_{0}}{\rho_{1}}+\sqrt{{\left(\frac{R_{2}S^{2}eC_{0}}{\rho_{1}}\right)}^{2}+\frac{2US^{2}eC_{0}}{\rho_{1}}}t.$$

Differentiating Equation (5) with respect to time gives the expression for the current:

(6) $$I(t)=\frac{U}{R_{2}}{\left(1+\frac{2U\rho_{1}}{R_{2}{}^{2}S^{2}eC_{0}}t\right)}^{-1/2}.$$

Note that a formula equivalent to Equation (6) was obtained earlier by Prieto and Liñares [35] and later extended by Oven [33] to three types of moving ions. It should be noted that we followed macroscopic description using only Ohm’s law and evident assumption that depth of the poling is proportional to the charge passed through the sample (see Equation (3)). We believe that this approach is more convenient for use since the current is expressed via macroscopic values as resistances and resistivities rather than microscopic mobilities. Although, if one expresses the resistivities in terms of mobilities, Equation (6) becomes identical to the ones in [33,35].

We should note the developed model describes well the results obtained in the present experiments, however, it may not be directly applicable to other poling techniques. Particularly, for poling with a needle corona discharge the front of the poling is rather spherical than plane, therefore, Equation (3) should be generalized via replacement I(t)/S→j(t,r), where j(t,r) is a coordinate-dependent current density. Penetration of other positively charged species from the atmosphere, which has also been recorded [16,36], may also limit the applicability of the developed model if the concentration of these species is high. In poling with pressed electrodes, depending on regimes, there may be a lack of positive ions in the environment, therefore, other ions inside the glass can be involved in motion, e.g., bivalent Ca^2+^, as was reported in [5]. Additionally, such poling suffers issues with repeatability because the peculiarities of pressing the electrodes to the specimen, on which the result strongly depends, are not amenable to precise control. 

Using the above model, we approximated the temporal dependencies of the poling current, which are presented in Figure 2a, with Equation (6). We took a general approximating function $I(t)=\frac{A}{\sqrt{1+Bt}}$, where *A* and *B* are fitting parameters. In calculations, the following parameters were used: *e* = 1.6 × 10^−19^ C, *C*_0_ = 6.9 × 10^27^ m^−3^ according to the glass composition, *S* = 0.64 cm^2^, *d*_2_ = 1.2 mm (1 mm for the specimen plus 0.2 mm for the covering glass), and only *ρ*_1,2_ were unknown values that were deduced from the fitting. In experimental dependencies, we cut off first several tens of seconds since the poling process is only establishing there, i.e., we used only descending part of the curves. 

**Figure 6:**
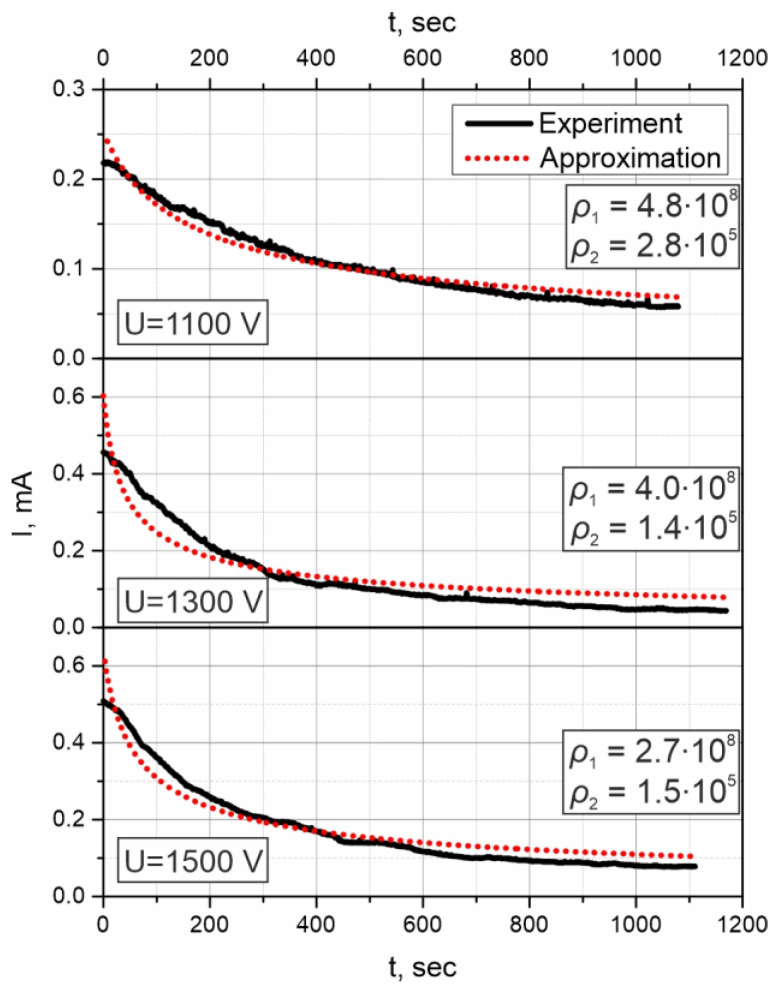
Polarization current dependencies on time (solid curves) for samples poled under 1100 V (**upper**), 1300 V (**middle**) and 1500 V (**lower**) and fitting curves (dotted). Values of resistivities *ρ*_1,2_ (in Ohm∙m) providing the best approximation are indicated near the curves.

The fitting curves are presented in Figure 6 together with the experimental ones, and the deduced values of *ρ*_1,2_ are indicated on the plots. The quality of approximations is satisfactory, and discrepancies may be caused by the impact in resistivity by the air gap the cathodic side of the specimen, for the pressing is not ideal. Calculating the average values and the standard deviations, we found: *ρ*_1_ = (3.8 ± 1.0) × 10^8^ Ohm∙m and *ρ*_2_ = (1.9 ± 0.8) × 10^5^ Ohm∙m. Expressing the mobilities of hydrogen/hydronium and sodium, *μ*_1,2_, via resistivities of the regions as $\mu ={\left(eC_{0}\rho \right)}^{-1}$, we obtained *μ*_1_ = (2.4 ± 0.8) × 10^−18^ m^2^V^−1^s^−1^ and *μ*_2_ = (4.8 ± 1.8) × 10^−15^ m^2^V^−1^s^−1^. We compare these results with the known literature data: in the classical work of Mehrer and coauthors [37], the value of Na^+^ diffusion coefficient *D_Na_* = 2 × 10^−16^ m^2^s^−1^ was obtained for the temperature 520 K and the glass with very similar composition. This corresponds to the mobility *μ*_Na_ = 5 × 10^−15^ m^2^V^−1^s^−1^, if the Haven ratio [38] is 1.0, however, the real mobility value is slightly lower, since the Haven ratio for soda-lime glasses is typically <1.0 [39]. Nonetheless, coincidence with our estimation for the mobility of sodium ions, *μ*_2_, is exceptional. Regarding hydrogen/hydronium mobility, there is lack of reliable experimental data. Though, common estimations give three orders less than Na^+^ mobility according to Oven [33] and 0.5 × 10^−3^ of Na^+^ mobility according to Doremus [40] that also strongly correlates with our results.

## 4. Conclusions

To sum up, we presented a technique of glass poling using resistive barrier discharge plasma in atmosphere. Measured polarization currents and SHG in the specimens of soda-lime glass poled under different voltages have demonstrated that poling is only efficient under voltages sufficient to ignite the plasma (about 1100 V in our case). The effect of poling under lower voltages is negligible. We attribute this to the fact that plasma discharge generates from atmospheric water vapor plenty of hydrogen/hydronium ions, capable of penetrating into the glass in poling. This is actually an open-anode regime of poling. For lower voltages, on the contrary, the number of hydrogen/hydronium ions in atmosphere is insufficient for the effective charge compensation, and the process cannot be considered as open-anode poling. Thus, we showed that the simple openness of the anode to atmosphere does not necessarily provides open-anode configuration of poling. We proposed a very simple model of open-anode poling which represents poling glass using an equivalent circuit of two series resistances and Ohm’s law. This allowed us to obtain the same expression for the polarization current as in earlier works, but represented via macroscopic parameters as resistivities and resistances. Fitting our measurements of polarization current with a theoretical expression, we revealed the resistivities of poled and unpoled regions, respectively: *ρ*_1_ = (3.8 ± 1.0) × 10^8^ Ohm∙m and *ρ*_2_ = (1.9 ± 0.8) × 10^5^ Ohm∙m. These correspond to hydrogen/hydronium and sodium ions mobilities *μ*_1_ = (2.4 ± 0.8) × 10^−18^ m^2^V^−1^s^−1^ and *μ*_2_ = (4.8 ± 1.8) × 10^−15^ m^2^V^−1^s^−1^, respectively. These values are in a very good agreement with the known literature data [33,40].
